# Latent profiles of psychological capital in clinical nursing teachers and their association with the practice environment of nursing and perceived social support

**DOI:** 10.3389/fpsyg.2025.1527252

**Published:** 2025-04-16

**Authors:** Yunling He, Dongxue Wang, Lining Wang, Rong Liao

**Affiliations:** ^1^Second People’s Hospital of Yibin, Yibin, China; ^2^Harbin Medical University (Daqing), Daqing, Heilongjiang, China

**Keywords:** clinical nursing teachers, psychological capital, practice environment of nursing, latent profile analysis, social support

## Abstract

**Background:**

Clinical nursing teachers (CNTs) play a critical role in nursing education, and their psychological capital (PsyCap) acts as an intrinsic motivational resource to assist them in facing the challenges of clinical teaching.

**Purpose:**

This research aims to examine the diversity of PsyCap in CNTs and its associated influencing factors via latent profile analysis.

**Methods:**

A cross-sectional study of 322 CNTs was conducted using four scales, following the STROBE statement guidelines.

**Results:**

The levels of PsyCap among CNTs could be categorized as low (24.1%), moderate (53.6%), and high (22.3%) PsyCap. Moreover, with the multiple logistic regression analysis, there are significant effects of the length of nursing experience, fertility status, self-reported health, nursing practice environment, and social support on the different categories of the PsyCap of CNTs (*p* < 0.05).

**Conclusion:**

The majority of the PsyCap of CNTs was at the moderate level, with obvious classification characteristics, which are influenced by multiple sociodemographic factors, e.g., length of nursing experience, fertility status, self-reported health, practice environment of nursing, and perceived social support.

## Introduction

As a key role in clinical teaching, clinical nursing teachers (CNTs) are essential for ongoing nursing education and training, the guidance of new nurses, and the practical training of nursing trainees. Through evidence-based pedagogical strategies such as bedside demonstrations and reflective case debriefings, CNTs cultivate students’ critical thinking and decision-making abilities and help students gain insights into future professional roles (Stenseth et al., 2025; An et al., 2025). CNTs are specialized nursing professionals who fulfill dual roles as clinicians and educators. They maintain active engagement in frontline clinical practice while concurrently fulfilling instructional responsibilities for nursing students’ clinical internships (Sun et al., 2025). Studies have indicated that 17–38% of nurses experience psychological distress, including emotional exhaustion and anxiety (Chen et al., 2019). The psychological well-being status of nurses has become a growing concern for healthcare institutions and communities as the medical field continues to evolve. Compared with regular nurses, CNTs are generally subjected to higher levels of job-related stress and suffer multiple stressors, such as interpersonal, career development, and role-related stresses (Lake et al., 2021; Xu et al., 2021; Sun et al., 2023). The psychological stress could heighten burnout levels among CNTs and diminish their teaching ability, which presents risks to the overall quality of healthcare delivery and knowledge transfer between CNTs and nursing students (Wu et al., 2021). Thus, it is necessary to pay more attention to the psychological well-being of CNTs.

Accompanied by the advancement of positive psychology and organizational behavior, there has been a growing emphasis on the influence of an individual’s inner capabilities on promoting mental well-being. Psychological capital (PsyCap) is a state of positive psychological development for individuals that includes four positive psychological resources: self-efficacy, optimism, hope, and resilience (Luthans et al., 2005; Luthans et al., 2007). Multiple studies implied that work-based PsyCap could produce positive results, and employees with high PsyCap levels will avoid negative emotions such as depression, stress, and anxiety and exhibit superior job satisfaction (Ngwenya and Pelser, 2021). Furthermore, PsyCap is vital for nurses to maintain a healthy mental state and manage workplace stressors, which could be regarded as an intrinsic drive to enhance nurses’ psychological and mental health, relieve occupational stress, and reduce nursing errors (Elliott and Fry, 2021). A high level of PsyCap is considered a motivational factor that enhances the workplace’s happiness and organizational performance (Elliott and Fry, 2021). In contrast, a lower level of PsyCap may aggravate negative professional emotions, such as compassion fatigue, burnout, and turnover intention (Labrague et al., 2020). Therefore, PsyCap, as a positive inner resource related to nurses’ mental health, nursing quality, and patients’ prognosis, has been widely considered by policy and management departments recently. As a dynamic and malleable construct, PsyCap plays a pivotal role in the daily work of CNTs (Lupșa et al., 2019). Effectively identifying the PsyCap levels of CNTs and their influencing factors is critical for nursing administrators to design targeted resilience-building interventions. Such initiatives empower CNTs to navigate mental health challenges and occupational adversities encountered in clinical settings, ultimately fostering sustainable professional well-being and optimizing pedagogical outcomes.

To date, some studies have identified the status quo and related factors of the PsyCap of nurses in specific countries or regions. A meta-analysis of 29 studies from 8 countries shows that the PsyCap of nurses is generally at a medium-high level (Yuan et al., 2022). The PsyCap of nurses in Asia was at the lowest level, which might be attributed to the heavy work difficulties they face (Turale and Nantsupawat, 2021). No prior study has investigated PsyCap in CNTs, which might exhibit distinct PsyCap characteristics. While interventions such as group cognitive-behavioral therapy and dialectical behavior therapy demonstrate PsyCap enhancement in nursing students, others have reported limited efficacy (Yan et al., 2025; Dong et al., 2024). Notably, the difference at the individual level is ignored by the majority of intervention programs aimed at promoting the PsyCap level of nurses. By identifying the potential profiles of PsyCap in CNTs and formulating targeted interventions, it may lead to a better intervention effect.

Latent profile analysis (LPA), a suitable method for uncovering the hidden categories of PsyCap at the individual level, can identify potential variations in CNTs’ PsyCap. This approach acts as a principle for grouping individuals into profiles based on their responses, reflecting similar professional characteristics, personal traits, or behavior patterns. This statistical analysis technique is effective and reliable in examining the mental health profiles of clinical nurses, including mental workload, professional fatigue, and emotional well-being among clinical nurses (Gu et al., 2023). The identification of different aspects of PsyCap in CNTs provides hospital managers with a new perspective on how to implement strategies to reduce negative physical and mental effects as well as improve the clinical performance of nursing staff. ‌To the best of our knowledge, currently, there is a lack of consistent criteria for categorizing PsyCap, and existing research primarily relies on scale scores without taking into account individual variations. Therefore, our study used the LPA method to identify distinct profiles of PsyCap among CNTs.

According to the job demands-resources theory, PsyCap can be affected by work and organizational factors; a good working environment can enhance the PsyCap of the followers, thus improving their work engagement (Baka, 2015). The practice environment of nursing is defined as the organizational characteristics of a nursing practice environment that promote or restrict professional nursing practice (Lake, 2002). The practice environment of nursing is a multi-factor structure composed of five characteristics: (i) nurses’ involvement in hospital affairs, (ii) the basis of nursing quality, (iii) the ability, leadership, and support of nurse managers, (iv) adequate staffing and resources, and (v) the relationship between doctors and nurses. Meanwhile, other studies have shown that a supportive organizational environment can develop PsyCap in nurses (Wang et al., 2024; Alhalal et al., 2024). Previous studies have only examined the association between the practice environment of nursing and PsyCap among regular nurses. Moreover, to the best of our knowledge, no previous study has focused on the relationship between the practice environment of nursing and the PsyCap profiles of CNTs. Thus, we examined the relationship between the practice environment of nursing and the PsyCap profiles of CNTs.

Previous studies have demonstrated that individuals with higher levels of perceived social support (PSS) tend to have higher levels of PsyCap (Lin et al., 2025; Fan et al., 2024). PSS was defined as an individual’s impression of whether he/she was supported by one’s social network (Barrera, 1986). According to the conservation of resources (COR) theory, social support serves as a potential resource. Through perceived social support, individuals can transform external social resources into internal psychological resources, thereby enhancing their PsyCap levels (Hobfoll, 1989). For example, a study found that a higher PSS level is associated with a higher level of self-efficacy (Lu et al., 2023), optimism (Bu et al., 2023), hope (Lu, 2022), and resilience (Zhang et al., 2022). Thus, we examined whether the PsyCap profiles of the CNTs were related to PSS.

Thus, we conducted a cross-sectional study to examine the level of PsyCap in CNTs, unveil the potential profiles of PsyCap, and examine the characteristics and factors linked to distinct PsyCap profiles. This study’s main hypotheses were as follows: (a) CNTs exhibit a moderate level of PsyCap; (b) PsyCap among CNTs can be classified into different subtypes; and (c) various demographic, social, and psychological factors, such as perceived social support, influence individuals in different profiles. The confirmation of these hypotheses is expected to reveal the multifaceted special features of PsyCap in CNTs, offering valuable perspectives based on positive psychology insights. Moreover, the findings of this study could inform hospital managers in implementing targeted support measures aimed at enhancing the PsyCap level of CNTs, thereby improving their psychological well-being and professional performance in teaching and practicing.

## Methodology

### Sample/participants

The mean estimation formula was used to determine the sample size based on the pre-survey, where *U_α_* = 1.96, *σ* = 0.75, and *δ =* 0.1. The formula is as follows (Ni et al., 2010).


$$N={\left(U_{\alpha }\;\sigma /\delta \right)}^{2}$$

The sample size was calculated using the mean estimation formula to be 216. Considering a 10% non-response rate, the final required sample size was calculated to be at least 259 cases.

A convenience sampling method was employed to conduct an online survey at three tertiary hospitals in Yibin City, Sichuan Province, China, from July 2023 to September 2023. A total of 341 questionnaires were gathered, and after excluding inadequate or redundant answers, 332 valid replies were gathered, yielding a response validity rate of 97.36%. The inclusion criteria were as follows: (1) Intermediate or senior title, with over 5 years of clinical nursing experience; (2) obtaining the qualification certificate of nurses and getting trained and certified in teaching; and (3) voluntary participation and signing of informed consent. We excluded the CNTs if they were unable to undertake clinical teaching within the past 6 months or were on leave (e.g., sick leave) during the survey.

### Measurement

#### Demographic questionnaire

The demographic questionnaire for this research comprised the following variables: gender, age, clinical experience (length of nursing experience, teaching years, and title level), marital status, fertility status, and self-reported health.

#### The practice environment scale of the nursing work index

The PES-NWI, developed by Lake (2002), was designed to evaluate the features of nurses’ practicing environments. Wang and Li (2011) adapted the content for the Chinese setting at a later stage. This scale comprises 5 dimensions with 31 items, each scored on a scale from 1 (very satisfied) to 4 (very dissatisfied). The overall score spanned from 31 to 124, with a higher score indicating a more favorable practice environment. The Cronbach’s *α* coefficient for this scale was 0.973.

#### Psychological capital questionnaires-reversed

The PCQ-R, developed by Luthans et al. (2007), was designed to assess the nurses’ PsyCap. Luo and He (2010) adapted the content for the Chinese setting at a later stage. This scale comprises 4 dimensions with 20 items, each scored on a scale of 1 (strongly disagree) to 6 (strongly agree). The overall score spanned from 20 to 120, where a higher score indicates a higher level of PsyCap. The Cronbach’s *α* coefficient for this scale was 0.968.

#### Multidimensional scale of perceived social support

The MSPSS, developed by Zimet et al. (1990), was designed to evaluate the social support encompassing family, friends, and intimate partners. Huang et al. (1996) adapted the content for the Chinese setting at a later stage. This scale comprises 3 dimensions with 12 items, each scored on a scale of 1 (strongly disagree) to 7 (strongly agree). The overall score spanned from 12 to 84, where a high number of points indicates a stronger perceived social support. The Cronbach’s α coefficient for this scale was 0.913.

### Data collection

The researchers employed an online questionnaire to gather data for the study. Before commencing the research, the researchers provided a detailed description of the objectives and methods to the head of the nursing department. Subsequently, an online survey was shared to CNTs through their managers. All participants received a thorough understanding of the study objectives, the participation method, and confidentiality importance before being invited to independently complete the survey. Only individuals who willingly consented were allowed to proceed with the questionnaire. To uphold data integrity, the survey was limited to a single response per Internet Protocol, with a completion time of approximately 7–10 min. Approval for this study was obtained from the Ethics Committee of the Second People’s Hospital of Yibin (No. 2023–142-01).

### Data analysis

The LPA analysis was carried out using Mplus 8.0, evaluating model fit with Akaike information criterion (AIC), Bayesian information criterion (BIC), sample-size adjusted BIC (aBIC), Lo–Mendell–Rubin likelihood ratio test (LMR-LRT), bootstrap likelihood ratio test (BLRT), and entropy. Lower AIC, BIC, and aBIC scores suggest a better model fit. *p*-values of < 0.05 for LMR-LRT and BLRT favor the k-category over the (k-1)-category models. A higher entropy near (1.0) indicates better classification. The models were compared to identify the best-fitting one.

The Statistical Package for the Social Sciences (SPSS), version 27.0, was used for the analyses in this study. Descriptive statistics, including frequencies and percentages for categorical variables and medians and quartiles for non-normally distributed continuous variables, were employed to analyze the demographic data. The chi-squared test and Kruskal-Wallis *H* test were used to compare variables between groups. Additionally, a multiple logistic regression analysis was conducted to investigate the factors influencing PsyCap among nursing students. A statistically significant difference was considered when *P* < 0.05.

## Results

### Participants characteristics

The study included 332 CNTs with ages varying between 25 and 55 years, of which 318 participants (95.78%) were female. A total of 131 teachers (39.46%) were specialty nurses. Regarding years in clinical teaching, 55.72 and 44.28% of teachers reported ≤10 years and more than 10 years, respectively. With respect to the fertility situation, 11.45, 80.42, and 8.13% of teachers reported being childless, having one child, and having ≥2 children, respectively. Among the CNTs, 99 (29.82%) had poor self-reported health, 117 (35.24%) had ordinary self-reported health, and 116 (34.94%) had good self-reported health. Additionally, the length of nursing experience for over half of the CNTs was over 10 years (69.88%). It is noteworthy that the majority of CNTs have an intermediate title level. Regarding the marriage status, 6.33, 84.94, and 8.73% of the CNTs were single, married, and divorced, respectively.

### Scores of the main variables

The PsyCap score was 100.00 (90.00, 107.00), while the practice environment of nursing score was 101.50 (93.00, 118.00) and the perceived social support score was 55.00 (52.00, 59.00). Additionally, we computed the scores for different dimensions of PsyCap, resulting in the following: the self-efficacy dimension scored 30.00 (27.00, 32.00), the hope dimension scored 30.00 (27.00, 32.00), the resilience dimension scored 15.00 (14.00, 17.00), and the optimism dimension scored 25.00 (23.00, 27.00).

### Latent profile analysis of PsyCap

**Figure 1:**
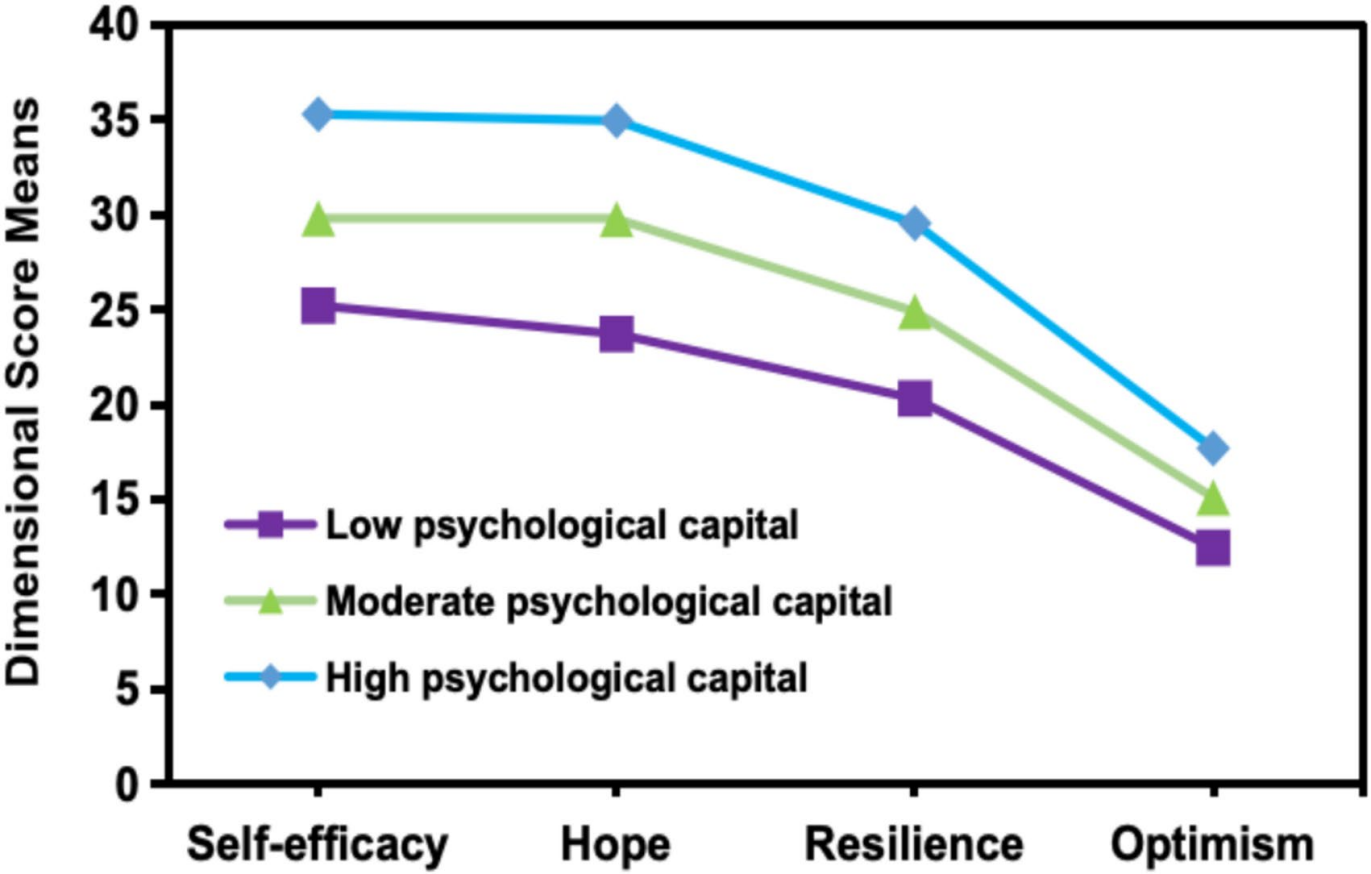
The latent profiles of psychological capital among clinical nursing teachers.

To identify the potential PsyCap profiles of CNTs, the one-profile model was initially used and then compared with models with an increasing number of profiles. The results in Table 1 indicate that all models exhibited a statistically significant BLRT *p*-value of less than 0.001, while the LMR test revealed significant results for both two and three profiles. Nevertheless, the LMR’s p-value for the fourth profile failed to reach significance. Therefore, it was excluded from further analysis because four or more profiles lacked representative validity. The AIC, BIC, and aBIC values gradually decreased as the number of profiles increased. In addition, because profile 3 had a higher entropy than profile 2, it was selected as the most suitable latent profile model. The distribution of the three-profile latent model across the four dimensions of PsyCap is shown in Figure 1.

**Table 1:** Clinical nursing teachers’ psychological capital latent profile model fitting indicators (*n* = 332).

Model	AIC	BIC	aBIC	LMR (*p*-value)	BLRT (*p*-value)	Entropy	Probability
1	7078.236	7108.677	7083.301	–	–	–	–
2	6507.237	6556.704	6515.467	<0.001	<0.001	0.906	0.29/0.71
3	5930.971	5999.464	5942.367	<0.001	<0.001	0.952	0.24/0.54/0.22
4	5849.753	5937.271	5864.314	0.110	<0.001	0.924	0.11/0.51/0.16/0.22
5	5815.820	5922.364	5833.574	0.028	<0.001	0.938	0.11/0.17/0.50/0.01/0/22

Profile 1, which included 24.1% of the participants, exhibited the lowest levels of PsyCap across all dimensions and was classified as having “low PsyCap.” Profile 2, having 53.6% of the participants, displayed median scores across all dimensions, indicating a “moderate level of PsyCap.” In Profile 3, 22.3% of the participants excelled in every dimension, earning the designation of “high PsyCap.”

### Factors associated with different profiles of PsyCap

The one-way analysis of variance showed statistically meaningful variations (*p* < 0.05) in age, fertility status, title of specialty nurse, length of nursing experience, self-reported health, practice environment of nursing, and perceived social support across the three profiles (Tables 2, 3). The identified significant factors were subjected to additional scrutiny, using the three PsyCap profiles as the dependent variables in the subsequent analysis. The profile labeled “Moderate PsyCap” was used as the baseline comparison reference for the analysis.

**Table 2:** Demographic results of clinical nursing teachers in different profile types [n (%), *N* = 332].

Variables	Low PsyCap	Moderate PsyCap	High PsyCap	*χ* ^2^	*P*
Sex				0.774	0.679
Male	3 (3.75)	9 (5.06)	2 (2.70)		
Female	77 (96.25)	169 (94.94)	72 (97.30)		
Age				12.050	**0.017**
≤30 years old	13 (16.25)	27 (15.17)	7 (9.46)		
31–44 years old	61 (76.25)	125 (70.22)	47 (63.51)		
≥45 years old	6 (7.50)	26 (14.61)	20 (27.03)		
Marital status				4.899	0.298
Single	2 (2.50)	11 (6.18)	8 (10.81)		
Married	72 (90.00)	150 (84.27)	60 (81.08)		
Divorced	6 (7.50)	17 (9.55)	6 (8.11)		
Fertility status				19.916	**0.001**
Childless	19 (23.75)	16 (8.99)	3 (4.05)		
One child	57 (71.25)	149 (83.71)	61 (82.43)		
≥2 children	4 (1.25)	13 (7.30)	10 (13.51)		
Specialty nurse				7.963	**0.019**
Yes	37 (46.25)	75 (42.13)	19 (25.68)		
No	43 (53.75)	103 (57.87)	55 (74.32)		
Title level				0.264	0.876
Intermediate	49 (61.25)	110 (61.80)	48 (64.86)		
Senior	31 (38.75)	68 (38.20)	26 (35.14)		
Length of nursing experience				22.516	**<0.001**
5–10 years	41 (51.25)	43 (24.16)	16 (21.62)		
>10 years	39 (48.75)	135 (75.84)	58 (78.38)		
Years of teaching				0.983	0.612
1–10 years	41 (51.25)	103 (57.87)	41 (55.41)		
>10 years	39 (48.75)	75 (42.13)	33 (44.59)		
Self-reported health				123.34	**<0.001**
Poor	53 (6.25%)	25 (14.04)	21 (28.38)		
General	18 (22.50)	95 (53.37)	4 (5.41)		
Good	9 (11.25)	58 (32.58)	49 (66.22)		

The bold values represent data with significant differences.

**Table 3:** Comparison of practice environment of nursing scores and perceived social support scores for each potential profile.

Variables	Low PsyCap (C1)	Moderate PsyCap (C2)	High PsyCap (C3)	*H*	*P*
Practice environment of nursing	93.00 (88.25, 96.00)	98.00 (93.00, 119.00)	122.00 (118.00, 124.00)	141.05	**<0.001**
Perceived social support	51.50 (45.00, 55.00)	54.00 (52.00, 57.00)	59.00 (56.00, 64.25)	63.396	**<0.001**

The bold values represent data with significant differences.

Title of specialty nurse (with yes as the reference), length of nursing experience (with ≤10 years as the reference), age (with ≥45 years old as the reference), fertility status (with ≥2 children as the reference), self-reported health (with good as the reference), practice environment of nursing, and perceived social support were employed as independent variables in a multiple logistic regression analysis.

The results revealed that fertility status, length of nursing experience, self-reported health, practice environment of nursing, and perceived social support were influencing factors of PsyCap in CNTs. Compared with the moderate PsyCap group, the probability of CNTs with a length of nursing experience >10 years was 8.134 times that of CNTs with less nursing experience (≤10 years) to be classified into the part of the high PsyCap group. CNTs with no child were 11.120 times (compared to CNTs with a child) more likely to be classified into the low PsyCap group. Furthermore, CNTs with poor self-reported health exhibited an increased likelihood of being classified into the low PsyCap group. Moreover, CNTs with higher levels of perceived social support and a more positive practice environment of nursing demonstrated greater probabilities of entering the high PsyCap group when compared with the moderate psychological group. The detailed results are shown in Table 4.

**Table 4:** Multiple logistic regression analysis of different latent profiles of psychological capital in clinical nursing teachers.

Variables		Low PsyCap group^a^				High PsyCap group^a^			
		B	SE	*P*	*OR* (95% CI)	B	SE	*P*	*OR* (95% CI)
Practice environment of nursing		−0.081	0.019	**<0.001**	0.922	0.232	0.038	**<0.001**	1.261
Perceived social support		−0.095	0.026	**<0.001**	0.910	0.146	0.036	**<0.001**	1.157
Specialty nurse		0.263	0.366	0.473	1.300	−0.920	0.547	0.093	0.399
Length of nursing experience: >10 years		−0.857	0.376	**0.023**	0.424	2.096	0.658	**0.001**	8.134
Age	<30 years old	−0.542	0.850	0.525	0.582	−1.499	0.985	0.128	0.223
	31–44 years old	0.206	0.617	0.738	1.229	−0.260	0.617	0.673	0.771
Fertility status	Childless	2.409	0.939	**0.010**	11.120	−1.607	1.217	0.187	0.201
	One child	0.843	0.756	0.265	2.322	−1.261	0.915	0.168	0.283
Self-reported health	Poor	2.663	0.513	**<0.001**	14.346	−0.501	0.628	0.425	0.606
	General	0.151	0.508	0.767	1.163	−3.882	0.819	**<0.001**	0.021

The bold values represent data with significant differences. ^a^Intergroup comparisons take the moderate PsyCap group as the reference group.

## Discussion

### Key findings

The level of PsyCap in CNTs was found to be medium-high in this study, which aligns with the results of a previous systematic review (Yuan et al., 2022). This research represents the first use of LPA in examining the PsyCap of CNTs, where homogeneous subgroups (latent profiles) among participants were identified to assess heterogeneity (Katja and Katariina, 2021). Contrasting traditional variable-driven methods, LPA employs a person-focused lens that highlights individuals and their distinctive qualities. This person-centered approach enables a more tailored and impactful customization of interventions or strategies based on a profound understanding of the unique traits within varying subgroups.

The heterogeneity of PsyCap was validated within the CNT population, categorizing into three distinct groups: low, moderate, and high PsyCap. The predictors of varied PsyCap profiles of CNTs were length of nursing experience, fertility status, self-reported health, perceived social support, and practice environment of nursing. In addition, CNTs with a moderate level of PsyCap comprised a majority share of the overall sample, which is similar to the findings of previous reports (Teng et al., 2023).

### Factors associated with different profiles of PsyCap

CNTs with > 10 years of nursing experience were found to have a higher tendency to be classified under the moderate or high PsyCap group in comparison with counterparts with 5–10 years of nursing experience. A scoping review revealed that younger nurses with fewer years of experience tend to exhibit lower levels of PsyCap, which can detrimentally impact them in nurturing positive work attitudes and refining clinical reasoning abilities, as well as diminishing their job satisfaction and adaptability (Okros et al., 2022). In general, CNTs with 5–10 years of nursing experience are the younger ones, facing more serious conflict between work-family and occupational stress, and are more likely to feel exhausted, thereby having a negative impact on PsyCap. With the increase in nursing experience, CNTs have refined professional skills and theoretical knowledge, a higher sense of responsibility, and a higher level of PsyCap (Sun et al., 2023).

Regarding fertility status, CNTs without a child were 11.120 times more likely to be classified into the low PsyCap group compared to the CNTs with a child, which could be attributed to relatively stable family environment and frequent emotional interactions associated with adulthood, which can enhance mental resilience and help mitigate negative career emotions. The CNTs with children had higher objective support, subjective support, and use of support than the CNTs without children, which can help improve their PsyCap (Zhang and Ni, 2024). In addition, when CNTs with children face higher work intensity, their spouses often take the initiative to take on household chores and child-rearing duties, effectively relieving their work pressure and psychological burden and enhancing the PsyCap (Zhang and Ni, 2024).

The self-reported health status reflects an individual’s perception of their overall social, biological, and psychological well-being. We found that CNTs with poor self-reported health exhibited a greater potential of being grouped in the low PsyCap group. Research has shown that good health, as indicated by self-reported data, is negatively associated with emotional exhaustion and work-related stress (Kung et al., 2021). Furthermore, previous research has suggested that good health is an individual asset for everyday life, which is negatively associated with burnout (Kung et al., 2021). Furthermore, a higher level of burnout is associated with a lower level of PsyCap (Meseguer de Pedro et al., 2021).

A satisfactory practice environment of nursing involves allowing nurses to participate in hospital affairs, the foundation of high-quality nursing care, the ability and leadership style of nursing managers, the cooperation between doctors and nurses, and sufficient manpower and material resources, which comprehensively reflects the features and nurses’ degree of satisfaction with the practice environment of nursing. Positive factors, above all, can effectively reduce occupational stress and improve psychological health (Song et al., 2023). In this study, we found that CNTs with better practice environments of nursing demonstrated greater probabilities of entering the high PsyCap group compared to those with poor practice environments of nursing. When CNTs perceived a more favorable nursing practice environment, their identification with work value strengthened and their sense of self-efficacy and hope for the future increased (Dan et al., 2023). In the meantime, they can cope with pressures and challenges with a more optimistic attitude and rapidly get rid of the negative emotions brought by setbacks and failures; at last, they have a higher level of PsyCap (Xiang et al., 2023). Pan et al. (2017) pointed out that nurses who were offered opportunities to participate in hospital affairs, reasonable payments, autonomy of working, and transformational leadership had a positive effect on their PsyCap and work engagement. Moreover, a previous study conducted with 541 pediatric nurses in tertiary hospitals demonstrated that the practice environment of nursing is positively related to PsyCap (Yan, 2023). A favorable nursing working environment can shape the positive psychological state of CNTs and improve overall work efficiency and teaching quality. Thus, nursing managers should pay attention to improving the practice environment of nursing, enhancing the PsyCap of CNTs.

PSS emerged as a set of support systems obtained by individuals through social interaction with other individuals, groups, and wider communities (Li et al., 2021). Our research revealed that CNTs with higher PSS levels demonstrated greater probabilities of joining the high PsyCap group compared with the moderate PsyCap group. As an important resource for nurses, PSS can strengthen nurses’ beliefs, help them actively cope with difficulties and challenges, and improve their resilience in the face of setbacks, playing a key role in maintaining the mental health of nurses in stressful clinical practice, while insufficient social support is related to the decline of mental health (Yun et al., 2024). The study suggested that, in a worsening nursing work environment, support from peers can reduce the pressure on nurses and jointly promote professional and psychological growth (Chisengantambu et al., 2017). Nurses with higher PSS are more likely to find efficient solutions in the face of stress and improve their PsyCap (Cao et al., 2021). Furthermore, empirical studies have demonstrated that PSS is positively related to psychological (Newman et al., 2014). In addition, a previous study suggested that both PSS and the organizational environment influence the PsyCap level (Peterson et al., 2011). According to the COR theory, social support serves as a potential resource that allows CNTs to transform external social resources into their internal psychological resources by appreciating social support, and their PsyCap level can be improved (Bickerton and Miner, 2023).

### Limitations

The study may be limited by its cross-sectional research design, which cannot establish causal relationships among variables. Future studies are recommended to employ a longitudinal research design to gain a more comprehensive understanding of the PsyCap of clinical nursing instructors. Furthermore, the use of convenience sampling in this research could potentially skew the accuracy of the results due to sample representation. To strengthen external validity, future research should consider adopting random sampling techniques that promote broader diversity and improved representation in sample selection. In addition, expanding the total sample size would further contribute to achieving this objective.

## Conclusion

The study used LPA to classify PsyCap into three distinct clusters among CNTs, unveiling the diverse characteristics of this demographic sample. These findings lay the foundation for developing targeted intervention programs centered on PsyCap, providing a road map for future research and practical applications. Nursing managers should focus on effectively identifying low PsyCap groups and implementing targeted measures to further enhance their PsyCap levels in CNTs by providing psychological training programs to help them cope with stress from both work and family. Furthermore, there may be an effective strategy to enhance the PsyCap among CNTs by implementing a targeted curriculum to improve the practice environment of nursing and social support.

## Data Availability

The raw data supporting the conclusions of this article will be made available by the authors, without undue reservation.
